# Not All Next Generation Sequencing Diagnostics are Created Equal: Understanding the Nuances of Solid Tumor Assay Design for Somatic Mutation Detection

**DOI:** 10.3390/cancers7030837

**Published:** 2015-07-17

**Authors:** Phillip N. Gray, Charles L.M. Dunlop, Aaron M. Elliott

**Affiliations:** Ambry Genetics, 15 Argonaut, Aliso Viejo, CA 92656 USA; E-Mails: cdunlop@ambrygen.com (C.L.M.D.); aelliott@ambrygen.com (A.M.E.)

**Keywords:** next generation sequencing, cancer panels, target enrichment, copy number, molecular inversion probes, somatic, germline

## Abstract

The molecular characterization of tumors using next generation sequencing (NGS) is an emerging diagnostic tool that is quickly becoming an integral part of clinical decision making. Cancer genomic profiling involves significant challenges including DNA quality and quantity, tumor heterogeneity, and the need to detect a wide variety of complex genetic mutations. Most available comprehensive diagnostic tests rely on primer based amplification or probe based capture methods coupled with NGS to detect hotspot mutation sites or whole regions implicated in disease. These tumor panels utilize highly customized bioinformatics pipelines to perform the difficult task of accurately calling cancer relevant alterations such as single nucleotide variations, small indels or large genomic alterations from the NGS data. In this review, we will discuss the challenges of solid tumor assay design/analysis and report a case study that highlights the need to include complementary technologies (*i.e.*, arrays) and germline analysis in tumor testing to reliably identify copy number alterations and actionable variants.

## 1. Introduction

Traditional methods for tumor characterization are tumor-type specific and include assays such as immunohistochemistry (IHC), *in situ* hybridization (ISH), quantitative PCR (qPCR), Sanger sequencing and gene signature microarrays [1,2,3,4,5,6,7,8]. These types of assays are highly specific and provide limited information. For example, the Roche Cobas qPCR platform has FDA approved *in vitro* diagnostic (IVD) products available for EGFR and BRAF, but the output is limited to either one mutational hotspot (BRAF V600) or mutations in a handful of exons (EGFR exons 18, 19, 20 and 21) and are only approved for specific cancer types [9,10]. Likewise, ISH assays provide limited information, as the region of interrogation is limited to the probe sequence [11,12]. In contrast, whole genome sequencing (WGS) of tumors using next generation sequencing (NGS) is an unbiased approach that provides extensive genomic information about a tumor. WGS can provide information both at the single nucleotide level, as well as detect structural variations such as large rearrangements, gross deletions and duplications [13,14,15,16]. Unfortunately, the cost of sequencing is still too high for routine clinical WGS of tumor specimens. An alternative approach is exome sequencing, but even this method is not cost effective due to the large amount of data required to detect low-level variants and time needed to analyze thousands of genes [13]. Targeted gene panels are currently the best option for tumor characterization, as they allow multiple genes to be analyzed and can provide enough depth of coverage to detect minor allele frequencies in a cost-effective manner [17,18,19,20].

Due to the gaining popularity of NGS based tumor testing, guidelines for detecting tumor variants have recently been established by the State of New York Health Department [21]. Based on these recommendations, samples should have enough sequence data for a minimum average coverage of 500× so that minor allele frequencies of 5% can be reliably detected. With current NGS platforms, the data yield is high enough for multiple samples to be barcoded and sequenced together (*i.e.*, multiplexed). However, the size of a targeted gene panel (*i.e.*, the number/size of genes targeted) will dictate the level of multiplexing that can still achieve an average of 500× coverage per sample (*i.e.*, large panels have low levels of multiplexing and small panels have high levels). In order for NGS diagnostics to be accurate and cost-effective, a balance must be reached between panel size and the level of multiplexing. In addition, coverage and its reliability as a quality metric can differ depending on whether amplification-based or probe-based enrichment is utilized [22].

When designing a complex tumor profiling test for high-throughput diagnostic use there are numerous considerations that must be taken into account compared to designing a research level assay. Likely, the most important component in testing in which treatment decisions are being made is turn-around-time. Oncologists need to start patients on treatment quickly, especially when the cancer is aggressive or refractory. Therefore, the assay needs to have a simplified wet lab work-flow as well as a variant reporting structure which makes it possible to deliver results to the clinician in a timely manner (~14 days). Another important component of diagnostic testing is the costs associated and the potential for insurance reimbursement. There are a lot of technologies available today such as DNA sequencing, RNA sequencing, epigenetic profiling, circulating tumor cell enumeration, *etc.* which together would provide a nice comprehensive snap-shot of one’s cancer, however routinely it is not economically feasible to achieve. Likewise, insurance companies are only willing to reimburse a certain amount for testing, which is not likely to change until more clinically relevant outcomes can be correlated with profiling results. In accordance, labs will ultimately need to navigate the regulatory pathway to FDA and Medicare approval. Again, this will take time to gather the appropriate data to illustrate the benefit in treatment outcomes based on comprehensive testing and off-label drug use.

Recently, an NGS cancer panel was approved by Palmetto (a contractor for the Centers for Medicare and Medicaid Services) for patients with advanced non-small cell lung cancer, however, patients must have previously tested negative for EGFR mutations, ALK rearrangements and ROS1 rearrangements through non-NGS methods (MolDX: NSCLC, Comprehensive Genome Profile Testing (L35896)). While this approval is a positive step forward, the requirement for previous testing is perplexing as the approval is based on a study by Drilon *et al.* that supports first-line profiling of lung adenocarcinomas using NGS gene panels as a more comprehensive and efficient strategy than non-NGS testing [23]. NGS gene panels are being leveraged in some basket trials such as the NCI-IMPACT (Molecular Profiling-Based Assignment of Cancer Therapy) and NCI-MATCH (Molecular Analysis for Therapy Choice), where enrollment in a trial is based on a molecular marker (*i.e.*, mutation) rather than tumor type. These trials will link tumor profiling data to patient outcomes and clinical/phenotypic data. Ideally, datasets generated from these basket trials should be deposited in databases available to researchers. There is little doubt over time NGS based cancer diagnostics will be a key component in guiding personalized drug selection and shift the paradigm in how clinicians treat patients.

## 2. Amplification-Based Enrichment Methods

**Figure 1:**
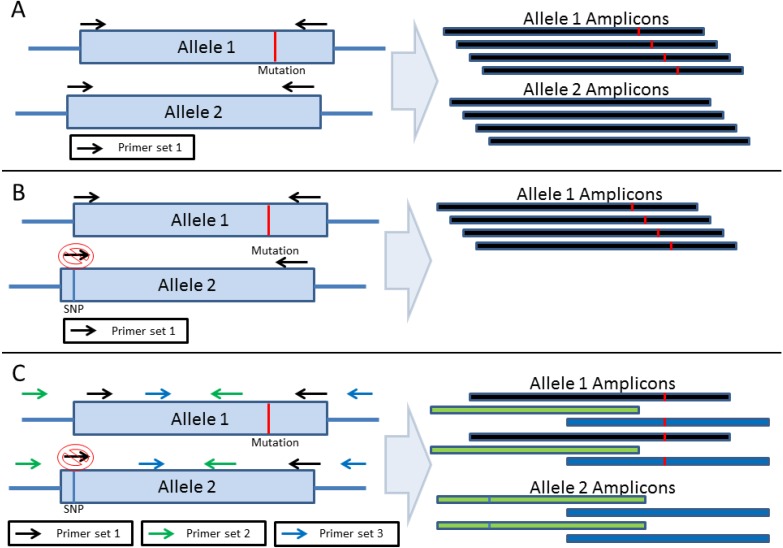
Amplification-Based Strategies for Target Enrichment. (**A**) Amplification based strategies for target enrichment consist of PCR amplification of the desired region using primers. (**B**) If a variant is located in the primer binding region, the primer may fail to bind resulting in failed amplification and allele dropout. (**C**) A tiled amplicon approach may be used to avoid allele dropout. In this scenario, multiple amplicons are generated for the same target minimizing allele dropout.

There are several commercially available amplification-based cancer panels such as the Ion Ampliseq™ Comprehensive Cancer Panel from Thermo Fisher/Life Technologies (Carlsbad, CA, USA), GeneRead Human Comprehensive Cancer Panel from Qiagen (Valencia, CA, USA) and Thunderbolts Cancer Panel from RainDance Technologies (Billerica, MA, USA). These platforms are based on the polymerase chain reaction (PCR) and are ideal when analyzing archived tissue that have limited material. Due to the relatively small size of primer sequences it is generally possible to design primers that do not amplify pseudogenes and can avoid most GC rich regions. Therefore, primer design strategies can generally achieve 100% coverage of a region of interest with minimal off-target sequence (Figure 1A) [22]. However, amplification-based target enrichment is susceptible to allele drop-out, which can result in false negatives. Allele drop-out occurs when a variant is located in a primer binding site and prevents primer hybridization, leading to failed amplification and allele bias [24,25,26]. Typically, allele drop-out is only detectable when the wild-type allele is affected, resulting in a suspicious homozygous mutation call (Figure 1B). The risk of allele drop-out can be reduced by a tiling primer design that results in multiple overlapping amplicons for each target (Figure 1C) [27]. This strategy is sufficient for germline DNA samples, but may not completely prevent allele drop-out in somatic cancer samples due to their heterogeneous nature. Primer design for germline samples can be guided by a reference sequence, so known single nucleotide polymorphisms (SNPs) can be avoided. DNA isolated from a population of somatic cancer cells will contain multiple acquired genotypes with varying allele frequencies. As these mutations can be present anywhere in the target region, allele drop-out may still occur if mutations are located in two adjacent primer binding sites.

**Figure 2:**
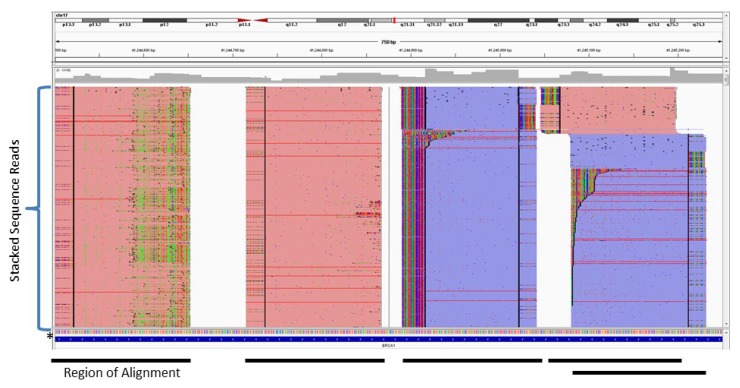
Sequence Read Alignment from an Amplification-based Enrichment Method. Individual sequence reads (red or blue horizontal lines) are stacked vertically and aligned to a reference sequence * at the base of the stack. The black bars on the bottom show the region of alignment and highlight the fact all sequence reads within a stack have the same start and stop positions.

As mentioned previously, amplicon-based enrichment strategies can achieve 100% coverage of a region with little off-target sequence as the start and stop coordinates of each amplicon are predetermined. A consequence of this selectivity is the true depth of coverage cannot be determined as PCR duplicates cannot be distinguished from amplicons generated from the original template. A PCR duplicate is a copy of a pre-existing amplicon and does not represent an independent data point [28]. PCR duplicates are typically identified and removed based on mapping coordinates [29,30,31]. However, all duplicate sequence reads for a particular target will have the same mapping coordinates as non-duplicates in amplicon-based enrichment strategies and thus cannot be removed. Figure 2 shows an example of NGS reads generated from an amplification-based method aligned to a reference sequence. The NGS reads are represented by horizontal red or blue bars that are stacked on top of each other. The level of stacking (*i.e.*, how many reads are stacked on a region) is the depth of coverage. Figure 2 shows a depth of coverage of >1000x (*i.e.*, there are more than 1000 reads covering each region), which includes PCR duplicates, and the reads are stacked perfectly in a column since each read has the same start and stop genomic coordinates.

**Figure 3:**
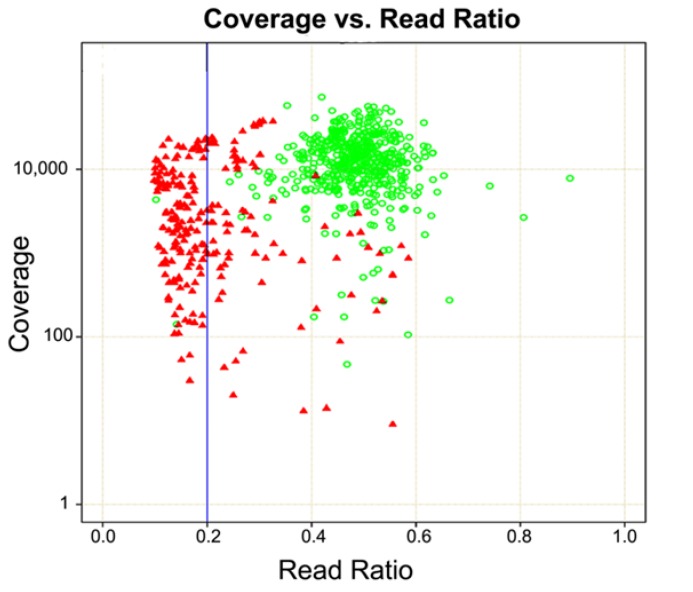
Coverage *vs.* Read Ratio for Amplification-based Enrichment of Germline Samples. The plot shows depth of coverage *vs*. observed allele ratios in NGS data. The green circles are Sanger confirmed variants and red triangles are false positives. Heterozygous alleles are not always represented in a 50:50 ratio in NGS data. As the plot shows, most true heterozygous calls range from ratios of 20:80 to 50:50 and alleles present below 20% (*i.e.*, 85:15, 90:10, 95:5, *etc.*) are typically false positives. Somatic samples may have true variants at 5%, so determining the true depth of coverage is critical for minimizing false positives.

The inability to identify and remove PCR duplicates results in increased false positives, as increased PCR duplicates inflate the false positive rate [32]. False positives are errors generated during amplification or sequencing and appear in the data as variants [33]. It is not uncommon to observe high false positive rates when analyzing germline samples at DNA inputs recommended for amplification-based enrichment methods [27,32]. The majority of these false positives can be eliminated by filtering out variants with an allele frequency less than 20% (Figure 3). The rate of PCR duplicates increases with decreased DNA input amounts [32]. This is relevant for FFPE tumor biopsy specimens as the number of PCR duplicates is expected to be higher due to (*i*) limited specimen material that restricts DNA input amounts, and (*ii*) poor sample quality that limits the amount of DNA that can be amplified [34]. These factors can pose a problem for heterogeneous tumor samples where detecting mutations with an allele frequency of 5% is desirable. A possible approach to identifying false positives is to run samples in duplicate, or split the PCR reaction into multiple tubes, but this increases sample handling and the risk of sample mix-up.

Single molecule tagging (SMT) was developed to identify PCR duplicates [35,36,37,38]. Smith *et al.* incorporated SMT technology into an amplification-based target enrichment protocol [32]. They looked at allele frequency differences for heterozygous SNPs from the same germline samples with and without PCR duplicates and observed a false positive rate of over 25% when duplicates were left in the data set. The false positive rate dropped to 5% after duplicate removal. The authors state the presence of PCR duplicates and corresponding false positive rate can give the appearance of a significant difference between samples when none exists. The SMT technology appears to be a viable option to identify PCR duplicates in amplification-based methods of tumor characterization, but may not be available or included in commercial panels or platforms.

## 3. Probe-Based Enrichment Methods

**Figure 4:**
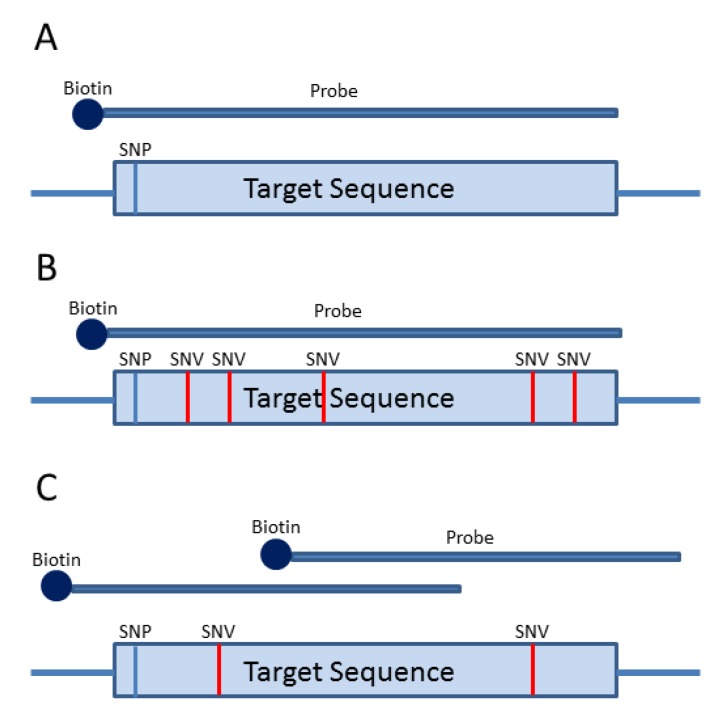
Probe-Based Strategies for Target Enrichment. (**A**) Probe-based strategies for target enrichment consist of generating biotinylated oligonucleotide probes (120 nucleotides in length) that are complementary to regions of interest. The size of the target will determine the number of probes required to capture the region. (**B**) Probe-based methods are not typically prone to allele dropout as the 120 nucleotide oligos can still hybridize in the presence of several (up to seven) mismatches. (**C**) Probe densities can be increased for regions that prove difficult to enrich.

Probe-based enrichment methods utilize long oligonucleotides complementary to a region of interest. The first published probe-based enrichment methods were based on microarray technology, where the probes were anchored to a solid surface [39,40,41]. Current probe-based platforms such as SureSelect from Agilent Technologies (Santa Clara, CA, USA), Lockdown Probes from Integrated DNA Technologies (Coralville, IA, USA) and SeqCap EZ from Roche-Nimblegen (Madison, WI, USA) are solution-based and use biotinylated oligonucleotide probes up to 120 nucleotides in length that are designed to capture a region of interest (Figure 4A). Since the probes are much longer than typical PCR primers, variants in the probe binding site typically do not affect hybridization to the target region and thus allele drop-out is not an issue (Figure 4B). This is important in tumor samples in which the mutation burden may be high. However, since probes can tolerate numerous mismatches in the target region, there is higher chance of enriching homologous regions (*i.e.*, pseudogenes), which can significantly increase off-target reads. Some genomic regions may be difficult to capture and require higher probe densities or tiled probes (Figure 4C). Other regions that are GC-rich or low complexity cannot be selectively isolated using probes [22]. Probe-based enrichment technologies hybridize to target regions contained within larger fragments of DNA. As a result, regions surrounding (or flanking) the target will be isolated and sequenced. This has the advantage of capturing neighboring regions of interest where probes cannot be designed, but will also isolate neighboring regions of no interest (*i.e.*, off target) that can reduce target region coverage if not minimized. Off-target sequence data can reduce overall coverage in regions of interest, which can be problematic when trying to detect low-level variants at high confidence in tumor samples.

**Figure 5:**
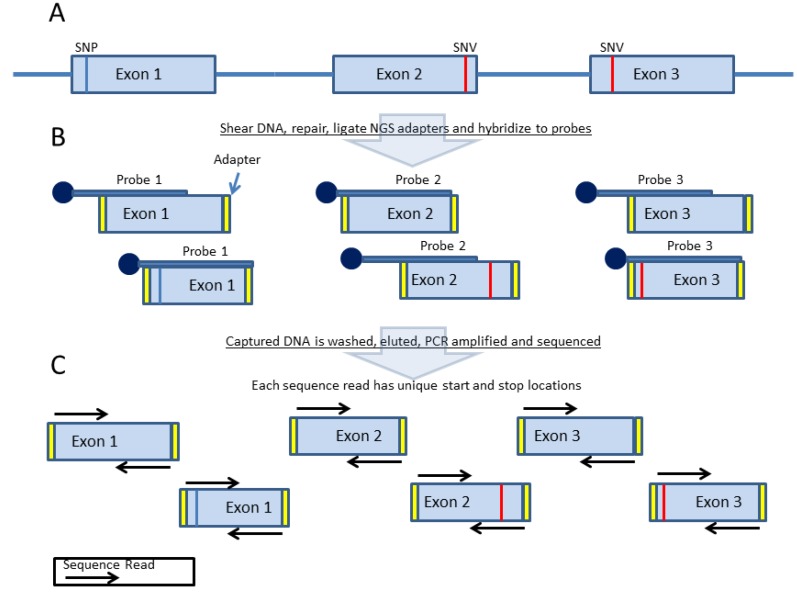
Sample Preparation for Probe-Based Target Enrichment. (**A**) DNA is randomly sheared, enzymatically repaired, NGS adapters ligated to the ends of fragmented DNA and PCR enriched. (**B**) Next, the NGS library is hybridized to biotinylated oligonucleotide capture probes. The size and nucleotide content of individual molecules of the same targeted region (*i.e.*, Exon 1 in the figure above) will differ due to random shearing. (**C**) The sequencing reads from these molecules will have unique start and stop coordinates when aligned to a reference, allowing identification and removal of PCR duplicates.

Unlike amplification-based enrichment methods, which can generate a sequence-ready library after just two rounds of PCR [27], most probe-based methods require traditional NGS library preparation (Figure 5A,B) [22]. Since DNA is randomly sheared for the NGS library prep, sequence reads for a particular target will have several different start and stop coordinates. Moreover, both ends of the DNA fragment are sequenced, so two independent reads with unique start and stop coordinates may be paired together generating a unique data point. As a result, PCR duplicates can be identified and removed from the dataset (Figure 5C), which reduces the false positive rate [32] and allows the true sequencing coverage depth to be determined.

**Figure 6:**
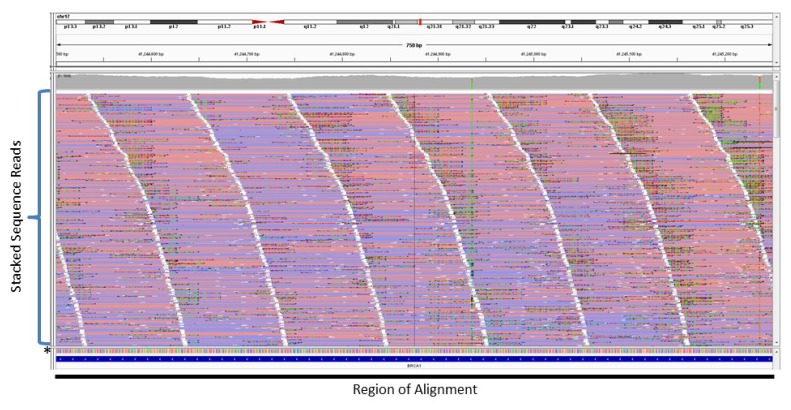
Sequence Read Alignment from a Probe-based Enrichment Method. Individual sequence reads (red or blue horizontal lines) stacked vertically and aligned to a reference sequence * at the base of the stack. The black bar on the bottom shows a continuous region of alignment with reads stacked in a staggered or step-wise manner. PCR duplicates have been removed, so reads within a stack will have unique start and stop coordinates.

Figure 6 shows an example of NGS reads generated from a probe-based enrichment method for a somatic DNA sample aligned to a reference sequence after PCR duplicate removal. The unique NGS reads are stacked on top of each other representing a depth of coverage of ~1200× for this particular sample.

The PCR duplicates for this somatic sample comprised approximately 50% of the sequence reads when using 1 µg input FFPE DNA in the enrichment process. Probe-based target enrichment requires more starting material than amplification-based enrichment, which could pose a problem if limited tumor biopsy material is available. However, as stated previously, PCR duplicates typically increase with decreased DNA input and the false positive rate is directly associated with PCR duplicate levels [32]. Importantly, in germline based assays all positives can be Sanger confirmed, so relaxed filtering can be used to avoid false negatives. In contrast, false positives are more of a concern for somatic samples since treatment may be based on the presence of a mutation and Sanger sequencing is not sensitive enough to detect low-level variants. Treatment may also be based on pertinent negative results, so false negatives are of potential concern.

## 4. Copy Number Analysis

While the initial application of NGS data was mutation analysis, computational tools have been developed to determine copy number variants (CNVs) using NGS data [42,43]. These tools are accurate when analyzing alleles from germline/diploid samples, but are limited when analyzing heterogeneous somatic samples, as they cannot confidently call low-level amplifications (*i.e.*, below 5×) [18]. Moreover, aneuploidy can be determined using NGS data from WGS [44,45], but cannot be readily detected using gene panels as the data only represents a fraction of the genome. In contrast, microarray-based platforms are highly sensitive and can detect single copy amplifications as well as aneuploidy and polyploidy [46,47]. Ploidy determination can have clinical significance and serve as a prognostic factor. In prostate cancer, DNA aneuploidy increases with stage and grade and can serve as a prognostic indicator for hormone therapy [48,49]. Also, aneuploidy of chromosome 17 is a prognostic indictor in breast cancer [50,51,52].

Array-based karyotyping provides a high-resolution genome-wide analysis of chromosome copy number and has become a standard clinical laboratory test for CNV determination [53,54,55]. Recently, Ciriello *et al.* analyzed data sets from approximately 3300 tumors from 12 tumor types characterized as part of The Cancer Genome Atlas [56]. They showed a bias in the type of mutations found in specific tumor types—specific tumors contained primarily either CNVs or SNVs/indels, but not both. Based on their analysis, breast and ovarian are two types of cancers driven primarily by CNVs. In fact, one of the best-known targeted therapies is Herceptin, which is prescribed, in part, based on HER-2/*neu* (ERBB2) copy number status [57]. Traditional methods for determining HER-2/*neu* status are IHC and ISH [58]. However, it is estimated that 20% of cases are either equivocal (due to IHC scores of 2+ and/or HER2/CEP17 ratios between 1.8 and 2.2) or discordant because IHC and ISH results contradict each other [59,60,61]. Hansen *et al.* [62] and Gunn *et al.* [60] analyzed breast cancer specimens to determine HER-2/*neu* status with a microarray-based approach and compared the results with those previously determined by IHC/FISH methods. Both groups showed the microarray method to be more sensitive and point out the discrepancies between the microarray method and FISH are due primarily to amplification or deletion of the chromosome 17 centromere region, which skews the HER2/CEP17 ratio. These studies highlight the advantage of using a global approach to determine copy number status as targeted diagnostics may lead to incomplete or incorrect results.

Microarray-based diagnostics have been in use for several years now and, similar to other DNA-based diagnostics, DNA quality will impact results. DNA extracted from FFPE specimens is typically degraded and damaged, and generating high quality microarray data can be challenging for this sample type [63]. The introduction of molecular inversion probes (MIPs) into the sample preparation process for array-based CNV and SNP analysis of FFPE specimens has greatly improved the data quality [64]. Unlike other array-based CNV analysis methods, MIP-based protocols only use the specimen DNA in the initial probe hybridization. As a result, the assay is not as sensitive to DNA quality as other array-based methods that use labeled specimen DNA to probe the array. Although there is an added cost associated with microarray analysis, coupling NGS with a microarray provides the most accurate and reliable genomic profile of a tumor.

## 5. Germline Status Incorporated into Bioinformatic Analysis

The bioinformatics pipeline utilized to detect tumor variants is likely the most important variable to consider when developing a somatic based assay as it can drastically affect the sensitivity, specificity and accuracy of the test. Several bioinformatics software programs exist for the analysis of somatic DNA [65,66]. Some programs perform tumor-only analysis while others, such as VarScan2 [67] and MuTect [68], perform paired analysis using NGS data from both tumor and matched normal tissue or blood. While a recent news story indicated most diagnostic labs perform tumor-only sequencing due to cost considerations [69], Jones *et al.* published a comparison of tumor only *vs.* tumor/germline paired analysis and found up to one-third of actionable mutations in tumor-only analysis are classified incorrectly as somatic when they are actually germline [70]. Actionable mutations are genomic alterations that may predict sensitivity or resistance to established or emerging therapies and include mutations that (*i*) have an FDA approved therapy in the patient’s tumor type; (*ii*) have an FDA approved therapy in a different tumor type (off-label); (*iii*) have a drug targeting the altered gene currently in a clinical trial; or (*iv*) provide prognostic information [17,20]. They observed germline mutations that are classified as actionable and have Cosmic IDs, including ERBB2/I1128V and MSH6/F726L. If these mutations are incorrectly classified as somatic, inappropriate treatment may be prescribed. Knowing the germline status of the patient can not only aid in the clinical management of the patient and their family members but also help correctly classify the variant as pathogenic or benign. For example, inherited mutations in oncogenes have only been described for a few genes [71]. Therefore, if an oncogene mutation is detected in the tumor and the germline of the patient, it is likely benign. In addition, the driver mutation of a tumor can be identified if it is determined that a particular pathogenic tumor suppressor mutation is germline. Likewise, if a mutation in a tumor suppressor such as BRCA1 or BRCA2 is only found in the tumor and not the germline the patient and family member cancer susceptibility can be managed appropriately. Although, the inclusion of incidental germline findings may complicate patient management for the oncologist [72], the data is evident that tumor-only analysis may lead to inappropriate treatment with significant effects on patient safety and healthcare costs.

## 6. Case Study

The sample described here, characterized at Ambry Genetics, details the power of not only differentiating germline derived mutations from tumor, but also performing microarray CNV analysis. A stage 4 prostate cancer specimen from a 43 year old individual (FFPE DNA) was sent to Foundation Medicine for analysis on FoundationOne^®^, a CLIA validated probe-based target enrichment tumor profiling test [18]. The FoundationOne clinical report lists genomic alterations that may provide treatment options for the ordering physician. The treatment options may include (*i*) FDA approved therapies in the patient’s tumor type; (*ii*) FDA approved therapies in a different tumor type; or (*iii*) potential clinical trials. The report also lists variants of unknown significance. The results of the test are listed in Table 1. The MITF amplification was the only actionable variant included in the report and Foundation Medicine listed a potential clinical trial for this CNV. No treatment options were given for the FOXP1 amplification, IKZF1 loss and TMPRSS2 rearrangement. Moreover, the MITF and FOXP1 amplifications were listed as equivocal, or ambiguous. A total of eight variants of unknown significance (VUS) were reported, including an ATM V2424G mutation, which is a known pathogenic mutation implicated in hereditary breast cancer [73,74,75,76]. ATM is part of the homologous recombination repair pathway [77,78,79], which is currently being targeted in the clinic with PARP inhibitors [80,81].

The specimen was also analyzed using a probe-based enrichment cancer gene panel developed by Ambry Genetics. The panel, TumorNext™, analyzes 142 genes implicated in somatic and hereditary cancers. The test is designed to include a matched blood sample that is analyzed in parallel, so the germline status of mutations can be determined. In addition, CNV analysis was performed using OncoScan®, a MIP-based microarray platform for CNV analysis from Affymetrix [82].

**Table 1:** Results from FoundationOne for a prostate cancer specimen.

Genomic Alterations Identified			
MITF amplification-equivocal	FOXP1 amplification-equivocal	IKZF1 loss	TMPRSS2 rearrangement intron 2
**Variants of Unknown Significance**			
ATM V2424G	BCOR K1260R	CHD4 K775R	EGFR loss
FAT1 N4492K	INHBA R229Q	PREX2 M1298L	RPTOR A862T

The FoundationOne gene panel is significantly larger than TumorNext, so a direct comparison of results was not possible. ATM, EGFR and MITF are the only genes listed in the Foundation Medicine report that are common to both NGS panels. TumorNext detected the ATM V2424G mutation and determined it to be germline (see below for CNV detection). Importantly, since this is a known hereditary pathogenic mutation and the individual is extremely young for prostate cancer, it is highly likely this is the driver mutation and not a VUS. As discussed previously, knowing the germline status of mutations can have an impact on treatment strategies. In fact, there is at least one PARP inhibitor clinical trial available for patients with advanced cancer and ATM mutations [83]. Also, a PARP inhibitor olaparib (Lynparza) has been approved to treat advanced ovarian cancer [84], so there is the potential for off-label use. These potential treatment options were missed by the FoundationOne test. Perhaps Foundation Medicine would have been alerted to these treatment options if they determined the germline status of this mutation.

**Figure 7:**
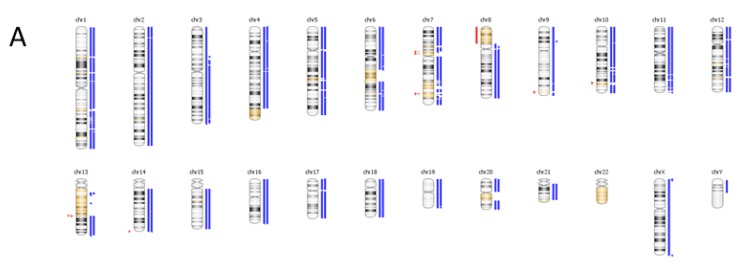

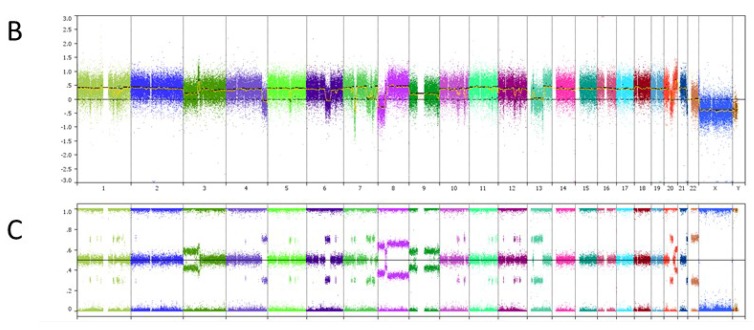
CNV Analysis using Affymetrix OncoScan. Karyotype (**A**), Log-ratio (**B**) and B Allele Frequency (**C**) plots showing polyploidy for a prostate cancer specimen.

The CNV results from OncoScan include a genome-wide karyotype, log-ratio and B-allele frequency plots (Figure 7). The karyotype plot shows the prostate tumor specimen is polyploidy, with almost all chromosomes amplified. The log-ratio plot shows most chromosomes are 4n and the B-allele frequency plot shows most chromosome regions have a balanced allele distribution (AA, AB and BB), which gives the overall impression of a diploid state. Most NGS bioinformatic pipelines include differences in read depth as a mechanism to determine CNV status. In this specimen almost the entire genome was amplified uniformly to 4n, so there are only a small number of regions where read depth differences can be distinguished. Thus, using an NGS based approach for CNV analysis in polyploidy samples is flawed. Practically every gene was amplified in this specimen and the FoundationOne report only lists two (both classified as equivocal), MITF and FOXP1, which are located in the same region of chromosome 3, with a copy number of 6n. Also, the FoundationOne report lists a loss of EGFR, which agrees with the OncoScan result. However, the OncoScan result shows a homozygous loss and the FoundationOne report does not specify whether it is homozygous or hemizygous. As mentioned previously, the ploidy state of prostate tumors has clinical significance and serves as a prognostic indicator [48,49].

## 7. Conclusions

NGS gene panels offer many advantages over traditional molecular assays currently used for tumor characterization and have become widely adopted. NGS gene panels allow multiple targets to be analyzed simultaneously, which may eliminate the need for reflex testing and preserves precious specimens by limiting the number of tests required for full characterization. As numerous tumor characterization NGS gene panels begin to enter the market it is important for users to understand the benefits and limitations of the methods and technologies being utilized. There are many nuances in designing NGS gene panels for solid tumor analysis and multiple options exist for each component of the workflow. Numerous factors can impact the accuracy, sensitivity and specificity of tumor sequencing including the target enrichment technology, sequencing platform and bioinformatics pipeline.

This review discussed the advantages of using a probe-based target enrichment strategy for NGS gene panels coupled with tumor/matched control paired analysis, which is critical to achieve a complete understanding of the significance of the detected alterations. The case study presented not only highlighted the advantages of incorporating germline analysis in somatic NGS gene panel assays, but also the limitations of using NGS panels to detect CNVs in somatic samples and the advantages of using arrays for this purpose. As more physicians leverage these tools for tumor characterization and become familiar with the technologies, best practices will emerge and become standardized.
